# Use of CYP3Ai and impact on outcomes in patients with acute myeloid leukemia treated with venetoclax plus azacitidine in the VIALE‐A study

**DOI:** 10.1002/ajh.26707

**Published:** 2022-09-16

**Authors:** Brian A. Jonas, Courtney DiNardo, Nicola Fracchiolla, Alexander Pristupa, Kenichi Ishizawa, Jie Jin, Marina Konopleva, Yishai Ofran, Pau Montesinos, Tibor Kovacsovics, Jun‐Ho Jang, Hagop Kantarjian, Yinghui Duan, Jalaja Potluri, Michael Werner, Keith W. Pratz

**Affiliations:** ^1^ Division of Hematology and Oncology, Department of Internal Medicine University of California Davis School of Medicine Sacramento California USA; ^2^ Department of Leukemia The University of Texas MD Anderson Cancer Center Houston Texas USA; ^3^ Hematology Unit Fondazione IRCCS Ca' Granda Ospedale Maggiore Policlinico Milan Italy; ^4^ Department of Hematology Ryazan Clinical Hospital Ryazan Russia; ^5^ Department of Internal Medicine III Yamagata University Faculty of Medicine Yamagata Japan; ^6^ Department of Hematology The First Affiliated Hospital, Zhejiang University College of Medicine Hangzhou Zhejiang China; ^7^ Department of Hematology, Shaare Zedek Medical Center, Faculty of Medicine Hebrew University of Jerusalem Jerusalem Israel; ^8^ Hematology Department Hospital Universitari i Politecnic la Fe Valencia Spain; ^9^ Division of Hematology and Hematologic Malignancies Huntsman Cancer Institute, University of Utah Salt Lake City Utah USA; ^10^ Samsung Medical Center Sungkyunkwan University School of Medicine Seoul South Korea; ^11^ AbbVie, Inc North Chicago Illinois USA; ^12^ Department of Medicine, Hematology‐Oncology Section Hospital of the University of Pennsylvania Philadelphia Pennsylvania USA

To the Editor:

Acute myeloid leukemia (AML) mainly affects older adults, and is associated with few treatment options and poor outcomes.^1^ The VIALE‐A trial (NCT02993523) investigated venetoclax (Ven), a potent B‐cell lymphoma 2 (BCL‐2) inhibitor, plus azacitidine (Aza) in patients with AML who were ineligible for intensive chemotherapy.^2^ The Ven + Aza combination demonstrated a median overall survival (OS) of 14.7 months compared with 9.6 months in the placebo (Pbo) + Aza arm (hazard ratio = 0.66; 95% confidence interval [CI]: 0.52–0.85, *p* < .001).^2^ Neutropenia and related infections are common in patients with AML, and antimicrobial prophylaxis for patients with AML is supported by data from randomized studies that investigated the use of levofloxacin and posaconazole in patients with neutropenia receiving high‐intensity chemotherapy.^3^, ^4^ The use of moderate or strong cytochrome P450 3A inhibitors (CYP3Ai) such as triazole antifungals, has been shown to increase Ven (a CYP3A substrate) exposure by reducing clearance, necessitating Ven dose reductions when concomitantly administered.^5^, ^6^ Thus, Ven dose modifications were required in VIALE‐A for the concomitant use of CYP3Ai.^2^ The purpose of this post hoc analysis of the VIALE‐A trial was to evaluate patient outcomes in the setting of Ven dose modifications due to concomitant use of CYP3Ai.

The randomized, double‐blind, placebo‐controlled phase 3 VIALE‐A trial evaluated the efficacy and safety of Ven + Aza compared with Pbo + Aza in patients with AML who were aged ≥75 years or ineligible for intensive chemotherapy. This post hoc analysis describes the OS, response rates, and rates of fungal infections in the setting of early CYP3Ai use when initiating Ven + Aza. The full VIALE‐A trial excluded patients with evidence of clinically significant uncontrolled systemic infection requiring therapy; the study design, statistical analysis, and results were previously reported.^2^ A summary of the study design and Ven dosing modifications for neutropenia can be found in the Supplement. In this analysis, early use of anti‐infective CYP3Ai is defined as medications initiated during Cycle 1 or 2 of Ven + Aza administration. Patients in VIALE‐A were divided into three groups according to their early use of CYP3Ai: no use of CYP3Ai (none), use of any moderate CYP3Ai (moderate), and use of any strong CYP3Ai (strong); moderate and strong CYP3Ai are listed in the Supplemental text. Seven patients in the Ven + Aza arm, and one patient in the Pbo + Aza arm received both moderate and strong CYP3Ais while on‐study and were included in both analysis groups. Echinocandin use as an alternative agent in cycles 1 and 2 was also evaluated. Unlike CYP3Ai, Ven dose modifications are not required for coadministration with echinocandins. Efficacy analysis was performed in the intent‐to‐treat (ITT) patient population (*N* = 431), which comprised 286 patients in the Ven + Aza arm, and 145 patients in the Pbo + Aza arm.^2^ The safety population comprised patients in the ITT population who received at least 1 dose of either Ven or Aza (282 patients in the Ven + Aza arm and 143 patients in the Pbo + Aza arm; *N* = 425). Descriptive analyses were performed to report summary statistics. Overall survival was analyzed using the Kaplan–Meier method. Response rates were defined as complete remission (CR), CR with incomplete hematologic recovery (CRi), and CR with partial recovery of peripheral blood counts (CRh) as a best response achieved anytime on study. Due to the exploratory nature of this post hoc analysis, no statistical comparisons were made.

The VIALE‐A trial randomized 433 patients from February 6, 2017 through May 31, 2019, and 431 were included in the ITT population.^2^ A total of 230 (80%) patients in the Ven + Aza arm of the ITT population received no CYP3Ai; 41 (14.3%) patients received moderate CYP3Ai and 22 (7.7%) received strong CYP3Ai. A similar proportion of patients in the Pbo + Aza arm (*N* = 145) received none (79.3%), moderate (12.4%), or strong (9.0%) CYP3Ai. Most patients who received CYP3Ai initiated the agent in Cycle 1, irrespective of treatment arm (Supplemental Table 1). Baseline characteristics were balanced across treatment arms and CYP3Ai subgroups (Supplemental Table 2) and have been previously reported.^2^

In the Ven + Aza arm, 67% (153/230) of patients who received no CYP3Ai, 61% (25/41) who received moderate CYP3Ai, and 64% (14/22) who received strong CYP3Ai had a best response of CR/CRi (Figure 1A). In the Ven + Aza arm, the median time to CR/CRi was 1.4 months in moderate and strong CYP3Ai subgroups, and 1.2 months in patients receiving none. A best response of CR/CRh was reached in 66% (151/230) of patients receiving no CYP3Ai, 54% (22/41) receiving moderate, and 64% (14/22) receiving strong (Figure 1B); median time to CR/CRh was 1.0, 1.0, and 1.1 months for patients receiving none, moderate, and strong CYP3Ai, respectively. The median OS of patients in the Ven + Aza arm who did not receive CYP3Ai in the first 2 cycles of therapy was 15.2 months (CI: 11.2–20.8), compared with 12.3 months (CI:7.6–19.3) and 12.2 months (CI: 3.9–21.1) in patients who received moderate and strong CYP3Ai, respectively (Figure 1C). Similar trends were observed for patients in the Pbo + Aza arm (Figure 1D). The median OS for patients who received echinocandins only (*n* = 26; 11.3%) in the first 2 cycles was 9.9 (6.4, NE) months compared with 12.2 (7.6, 19.3) months in patients who received either strong or moderate CYP3Ai (Supplemental Table 3).

**FIGURE 1:**
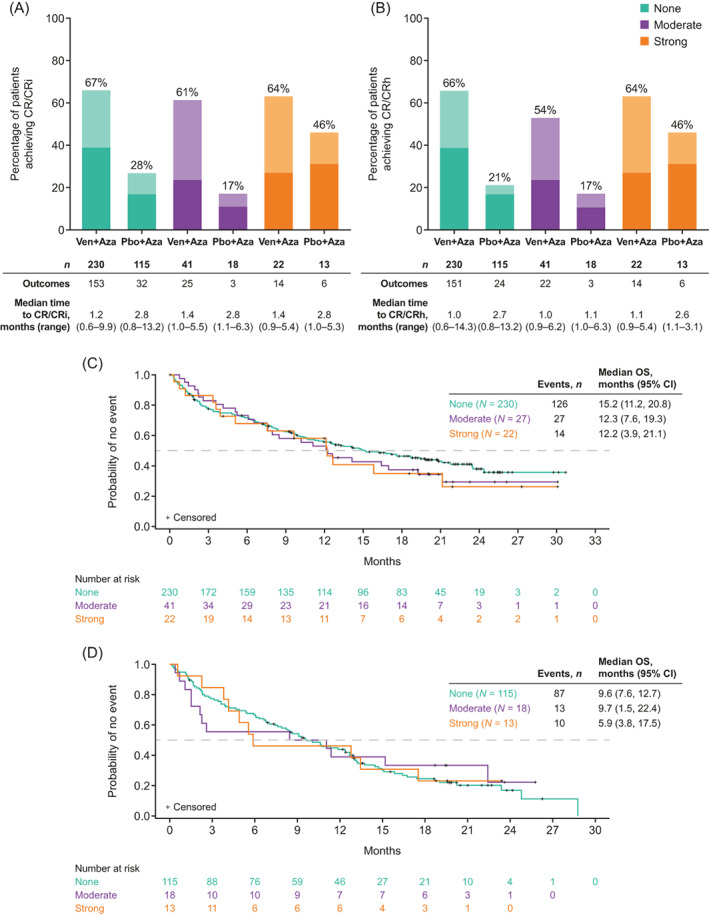
Response Rates and overall survival in patients receiving none, moderate, and strong CYP3Ai Initiated in cycle 1 or cycle 2 concomitant with venetoclax + azacitidine (ITT population). Dark shading represents CR, light shading represents CRi/CRh. (A) Summary of CR/CRi in patients receiving none, moderate, and strong CYP3Ai inhibitors; (B) Summary of CR/CRh in patients receiving none, moderate, and strong CYP3Ai inhibitors; (C) OS in the Ven + Aza arm among patients who received none, moderate, and strong CYP3Ai; (D) OS in the Pbo + Aza arm among patients who received none, moderate, and strong CYP3Ai. Aza, azacitidine; CI, confidence interval; CR, complete remission; CRh, CR with partial recovery of peripheral blood counts; CRi, CR with partial hematologic recovery; CYP3Ai, cytochrome P450 3A inhibitors; ITT, intention‐to‐treat; OS, overall survival; Pbo, placebo; Ven, venetoclax

Among patients in the Ven + Aza arm of the safety population, 226 received no concomitant CYP3Ai, 41 received moderate CYP3Ai, and 22 received strong CYP3Ai in the first 2 cycles of therapy. Rates of grade 3/4 infections were 44% (18/41) in the moderate CYP3Ai subgroup and 36% (8/22) in the strong CYP3Ai subgroup compared with 33% (74/226) in patients receiving no CYP3Ai (Supplemental Table 4). Fungal infections ≥ grade 3 occurred at rates of 10% (4/41) in the moderate subgroup, 9% (2/22) in the strong subgroup, and 2% (4/226) in the no CYP3Ai group.

In this post hoc analysis of the VIALE‐A trial, approximately 20% of patients received moderate and strong CYP3Ai (with recommended Ven dose reductions) and did not appear to have different OS or composite remission rates (CR/CRi or CR/CRh) compared with patients not receiving CYP3Ai agents. The frequency of infections among patients who received moderate and strong CYP3Ai did not appear different from those patients who did not receive CYP3Ai. There are some inherent biases in these analyses in that participating investigators were not required to document the reason for CYP3Ai administration, and “early use” was adjudicated using a clinical database in a post hoc fashion, so it was not possible to definitively determine whether the inhibitors were used for prophylaxis; although the intention was to identify prophylactic use only, CYP3Ai administered to treat infections arising subsequent to enrolment cannot be ruled out. The occurrence of reverse causality (i.e., patients with a higher risk of infection being more likely to receive CYP3Ai) also cannot be excluded.

As VIALE‐A was not designed to investigate the impact of CYP3Ai use on survival, response, or rate of infections, and considering the small number of patients receiving these agents, additional studies are warranted to confirm any relationships seen in this analysis. Although the frequency of infections did not decrease with CYP3Ai use, invasive fungal infections were uncommon overall and were similar between patients who received CYP3Ai agents and those who did not. The long‐lasting effect of Ven dose reduction with prolonged concomitant strong CYP3Ai needs to be addressed in larger studies. However, in the context of this post hoc analysis, in which statistical comparisons were not appropriate, these data suggest that using concomitant moderate or strong CYP3Ai with appropriate Ven dose reductions for patients with AML may not impair remission achievement or survival.

## AUTHOR CONTRIBUTIONS

*Study conception and design*: Brian A. Jonas, Courtney DiNardo, Yinghui Duan, Jalaja Potluri, Michael Werner, and Keith Pratz. *Collection and assembly of data*: All authors. *Analysis and interpretation of data*: All authors. *Manuscript writing, editing, and approval*: All authors.

## FUNDING INFORMATION

Venetoclax is being developed in a collaboration between AbbVie and Genentech. AbbVie and Genentech funded this study (NCT02993523) and participated in the study design, research, analysis, data collection, interpretation of data, reviewing, and approval of the publication. All authors had access to relevant data and participated in the drafting, review, and approval of this manuscript. No honoraria or payments were made for authorship. Medical writing support was provided by Rachel M. Richardson, PharmD, MS, employee of AbbVie, and Hayley Ellis, PhD, of Fishawack Communications Ltd, funded by AbbVie.

## CONFLICT OF INTEREST

Brian A. Jonas: Consultancy/advisory role for AbbVie, BMS, Genentech, Gilead, GlycoMimetics, Jazz, Pfizer, Servier, Takeda, Tolero, and Treadwell; protocol steering committee for GlycoMimetics; data monitoring committee for Gilead; travel reimbursement from AbbVie; research funding to institution from 47, AbbVie, Accelerated Medical Diagnostics, Amgen, AROG, BMS, Celgene, Daiichi Sankyo, F. Hoffmann‐La Roche, Forma, Genentech/Roche, Gilead, GlycoMimetics, Hanmi, Immune‐Onc, Incyte, Jazz, Loxo, LP Therapeutics, Pfizer, Pharmacyclics, Sigma Tau, and Treadwell. Courtney DiNardo: Consultant and on Advisory Boards for AbbVie, Agios, Aprea, Celgene/BMS, ImmuneOnc, Kura, Novartis, Takeda, Notable Labs; research support provided to her institution by AbbVie, Agios, Calithera, Cleave, BMS/Celgene, Daiichi‐Sankyo, ImmuneOnc, Loxo. Nicola Fracchiolla: Honoraria from Amgen, Pfizer, Gilead, and AbbVie; speakers' bureau for Amgen, Pfizer, and Gilead; board of directors or advisory committee membership with Amgen, Pfizer, Gilead, and AbbVie; travel accommodations and expenses from Amgen, Pfizer, Gilead, and AbbVie. Alexander Pristupa: Employment with State Institution of Health of the Ryazan Regional Clinical Hospital; speakers' bureau with Takeda and Pfizer; research funding from Takeda, Pfizer, Janssen, AbbVie, Daiichi Sankyo, Paraxel, Acerta, Pharmacyclics, Geron, and Beigene; honoraria from Takeda, Pfizer, Janssen, AbbVie, Daiichi Sankyo, Paraxel, Acerta, Pharmacyclics, Geron, and Beigene. Kenichi Ishizawa: Honoraria from Takeda, Ono, Chugai, Eizai, Novartis, and Celgene; research funding from SymBio, Bayer, AbbVie, and Novartis. Jie Jin and Jun‐Ho Jang: Nothing to disclose. Marina Konopleva: Consultancy/advisory role with AbbVie, Genentech, F. Hoffmann La‐Roche, Stemline Therapeutics, Forty‐Seven, Amgen, and Kisoji; research funding from AbbVie, Genentech, F. Hoffmann La‐Roche, Stemline Therapeutics, Forty‐Seven, Eli Lilly, Cellectis, Calithera, Ablynx, Agios, Ascentage, AstraZeneca, Rafael Pharmaceutical, and Sanofi; royalties, stock, and other options from Reata Pharmaceutical Inc.; patents and royalties with patent US 7795305 B2 on CDDO‐compounds and combination therapies, licensed to Reata Pharmaceutical. Yishai Ofran: Consultancy/advisory role for Pfizer, AbbVie, Novartis, Astellas, and Roche; board of directors or advisory committee membership with AbbVie, Novartis, and Pfizer. Pau Montesinos: Consultancy/advisory role for Celgene, Pfizer, and AbbVie; research funding from Pfizer, AbbVie, and Daiichi Sankyo; speakers' bureau with Astellas, Novartis, and Janssen. Tibor Kovacsovics: Honoraria from Agios, Astella, and Jazz; research funding from AbbVie, Glycomimetics Novartis, and Pfizer. Jun‐Ho Jang: Honoraria from Novartis and Amgen. Hagop Kantarjian: Research funding from AbbVie, Amgen, Ascentage, Bristol Myers Squibb, Daiichi‐Sankyo, Immunogen, Jazz, Novartis; honoraria from AbbVie, Amgen, Ascentage, Astellas, Astrazeneca, Biologix, Curis, Ipsen Biopharmaceuticals, KAHR Medical, Novartis, Pfizer, Precision Biosciences, Shenzhen Target Rx, and Taiho Pharma Canada. Yinghui Duan, Jalaja Potluri and Michael Werner: AbbVie employees and may own stock or stock options. Keith W. Pratz: Consultancy/advisory role for AbbVie, Astellas, Boston BioMedical, Bristol Myers Squibb, Novartis, Jazz Pharmaceuticals, Servier, and Celgene; research funding from AbbVie, Agios, Daiichi Sankyo, and Millennium.

## Supporting information


**Appendix S1** Supporting information.

## Data Availability

AbbVie is committed to responsible data sharing regarding the clinical trials we sponsor. This includes access to anonymized, individual, and trial‐level data (analysis data sets), as well as other information (e.g., protocols, clinical study reports, or analysis plans), as long as the trials are not part of an ongoing or planned regulatory submission. This includes requests for clinical trial data for unlicensed products and indications. These clinical trial data can be requested by any qualified researchers who engage in rigorous, independent, scientific research, and will be provided following review and approval of a research proposal, Statistical Analysis Plan (SAP), and execution of a Data Sharing Agreement (DSA). Data requests can be submitted at any time after approval in the United States and Europe and after acceptance of this manuscript for publication. The data will be accessible for 12 months, with possible extensions considered. For more information on the process or to submit a request, visit the following link: https://www.abbvie.com/our-science/clinical-trials/clinical-trials-data-and-information-sharing/data-and-information-sharing-with-qualified-researchers.html.
