# Supplementary material for: Use of CYP3Ai and impact on outcomes in patients with acute myeloid leukemia treated with venetoclax plus azacitidine in the VIALE‐A study
Source: Am J Hematol. 2022 Sep 16;97(11):E422–5. doi: 10.1002/ajh.26707 (PMC10286746; doi:10.1002/ajh.26707)
Supplement: Supplementary file 1 — Appendix S1 Supporting information. [file AJH-97-E422-s001.docx]

**Supplemental Appendix
Use of CYP3Ai and impact on outcomes in patients with AML treated with venetoclax plus azacitidine in the VIALE-A study**

**Table of Contents**

**Supplemental Text**

Summary of VIALE-A Patient Population

**Supplemental Tables**

Supplemental Table 1. CYP3Ai initiation during Cycle 1, Cycle 2, and subsequent cycles
Supplemental Table 2. Baseline characteristics (ITT population)
Supplemental Table 3. OS of patients receiving echinocandins and CYP3Ai initiated in cycle 1 or cycle 2 concomitant with venetoclax + azacitidine (ITT population)

Supplemental Table 4. Adverse events occurring in the first 2 cycles during which CYP3Ai use was evaluated (safety population)

**Supplemental text**

**Summary of VIALE-A Patient Population**

Eligible participants (N=433) were randomly assigned 2:1 to receive either venetoclax (Ven) 400 mg by mouth, daily, on Days 1–28 plus azacitidine (Aza) 75 mg/m^2^, subcutaneously or intravenously on Days 1–7 on a 28-day cycle (n=287), or a placebo plus Aza (n=146). Overall survival (OS) was the primary endpoint, and patients were stratified by age, cytogenetic risk, and geographic location. Two of the 433 patients randomized did not undergo stratification by cytogenic risk. Therefore, a total of 286 patients in the Ven + Aza arm and 145 patients in the placebo (Pbo) + Aza arm were included in the intent-to-treat population for efficacy analyses in VIALE-A. A total of 282 patients in the Ven + Aza arm and 143 patients in the Pbo + Aza arm received at least 1 dose of the study treatment and therefore were included in the safety analysis. Previously defined key secondary endpoints relevant to the current analysis are complete remission (CR), CR with partial hematologic recovery (CRh), and CR with incomplete hematologic recovery (CRi).

Strong CYP3Ai included: clarithromycin, itraconazole, posaconazole, and voriconazole. Moderate CYP3Ai included: ciprofloxacin, ciprofloxacin hydrochloride, clotrimazole, erythromycin, fluconazole, isavuconazonium, isavuconazonium sulfate, and levofloxacin lactate plus sodium chloride.

Per the VIALE-A protocol, concomitant medication was required for patients with an absolute neutrophil count of <500/µL. Protocol-mandated dose adjustments of Ven were 200 mg per day for moderate CYP3A4 inhibitors and 50 mg per day for strong inhibitors.

**Supplemental Tables**

**Supplemental Table 1.** CYP3Ai initiation during Cycle 1, Cycle 2, and subsequent cycles

|  | **Venetoclax + azacitidine**  **N=286** | | | **Placebo + azacitidine**  **N=145** | | |
| --- | --- | --- | --- | --- | --- | --- |
| **CYP3Ai Category*** | **Moderate^†^**  **(n=41)** | **Strong^†^**  **(n=22)** | **Any**  **(n=56)** | **Moderate^†^**  **(n=18)** | **Strong^†^**  **(n=13)** | **Any**  **(n=30)** |
| Initiated in Cycle 1, n (%) | 30 (73) | 19 (86) | 44 (79) | 16 (89) | 13 (100) | 28 (93) |
| Initiated in Cycle 2, n (%) | 10 (24) | 2 (9) | 12 (21) | 2 (11) | 0 | 2 (7) |
| Initiated after Cycle 2, n (%) | 19 (46) | 7 (32) | 23 (41) | 7 (39) | 3 (23) | 9 (30) |

Percentages are calculated using the number of patients receiving CYP3Ai in each category as the denominator.

*CYP3Ai category here describes the analysis groups defined as use at any time on-study. **^†^**Categories are not mutually exclusive; some patients received both moderate and strong CYP3Ai.
CYP3Ai, cytochrome P450 3A inhibitors.

**Supplemental Table 2.** Baseline characteristics (ITT population)

|  | **Venetoclax + azacitidine**  **N=286** | | | **Placebo + azacitidine**  **N=145** | | |
| --- | --- | --- | --- | --- | --- | --- |
| Duration of CYP3Ai agent use, median (range), days* | 12.5 (1–614)^†^ | | | 15.0 (1–731)^†^ | | |
| **CYP3Ai Category^‡^** | **None**  **(n=230)** | **Moderate**  **(n=41)** | **Strong**  **(n=22)** | **None**  **(n=115)** | **Moderate**  **(n=18)** | **Strong**  **(n=13)** |
| Age, median (range), years | 76  (49–91) | 76  (53–84) | 73  (62–84) | 76  (61–90) | 77  (60–86) | 75  (67–87) |
| De novo AML, n (%) | 179 (77.8) | 25 (61.0) | 15 (68.2) | 87 (75.7) | 14 (77.8) | 10 (76.9) |
| ECOG performance status, n (%)  0–1  2–3 | 125 (54.3)  105 (45.6) | 26 (63.4)  15 (36.6) | 11 (50.0)  11 (50.0) | 64 (55.7)  51 (44.3) | 11 (61.1)  7 (38.9) | 7 (53.9)  6 (46.2) |
| Cytogenetic risk, n (%)  Intermediate  Poor | 153 (66.5)  77 (33.5) | 21 (51.2)  20 (48.8) | 11 (50.0)  11 (50.0) | 76 (66.1)  39 (33.9) | 9 (50.0)  9 (50.0) | 5 (38.5)  8 (61.5) |
| CTC grade of neutropenia at study entry, n (%)  2  3  4 | 15 (6.5)  45 (19.6)  122 (53.0) | 4 (9.8)  2 (4.9)  28 (68.3) | 1 (4.5)  2 (9.1)  13 (59.1) | 11 (9.6)  23 (20.2)  48 (42.1) | 2 (11.1)  3 (16.7)  9 (50.0) | 1 (7.7)  4 (30.8)  4 (30.8) |

*Duration of any CYP3Ai use starting during cycle 1 or cycle 2. A patient may have multiple uses. ^†^For any end date that was not available, the end date was the date of study data cut. ^‡^Some patients received both moderate and strong CYP3Ai.
AML, acute myeloid leukemia; CTC, Common Toxicity Criteria; CYP3Ai, cytochrome P450 3A inhibitors; ECOG, Eastern Cooperative Oncology Group.

**Supplemental Table 3:** Overall survival of patients receiving echinocandins and CYP3Ai initiated in cycle 1 or cycle 2 concomitant with venetoclax + azacitidine (ITT population)

|  | **Echinocandins**  **only**  **(n= 26)** | **Moderate/strong CYP3Ai**  **only**  **(n=40)** |
| --- | --- | --- |
| Events, n (%) | 15 (57.7) | 26 (65.0) |
| Median overall survival, months (95% CI) | 9.9 (6.4, NE) | 12.2 (7.6, 19.3) |
| 6-month survival estimate (95% CI)  12-month survival estimate (95% CI)  24-month survival estimate (95% CI) | 80.3% (58.9, 91.3)  43.5% (23.8, 61.6)  39.1% (20.3, 57.6) | 69.9% (53.1, 81.7)  54.4% (37.7, 68.3)  30.3% (15.8, 46.1) |

CI, confidence interval; CYP3Ai, cytochrome P450 3A inhibitors; ITT, intention-to-treat; NE, not estimable.

**Supplemental Table 4.** Adverse events and deaths occurring in the first 2 cycles during which CYP3Ai use was evaluated (safety population)

|  | **Venetoclax + azacitidine**  **N=282** | | | **Placebo + azacitidine**  **N=143** | | |
| --- | --- | --- | --- | --- | --- | --- |
| **CYP3Ai Use*** | **None**  **(n=226)** | **Moderate (n=41)** | **Strong**  **(n=22)** | **None**  **(n=113)** | **Moderate (n=18)** | **Strong**  **(n=13)** |
| Any infection or infestation, n (%) | 119 (53) | 31 (76) | 14 (64) | 45 (40) | 12 (67) | 6 (46) |
| Grade 3/4 | 74 (33) | 18 (44) | 8 (36) | 31 (27) | 8 (44) | 4 (31) |
| Serious | 54 (24) | 21 (51) | 10 (45) | 25 (22) | 8 (44) | 5 (38) |
| Led to discontinuation | 26 (12) | 5 (12) | 4 (18) | 8 (7) | 0 (0) | 1 (8) |
| **Any invasive fungal infection, n (%)** | 6 (3) | 5 (12) | 2 (9) | 0 (0) | 0 (0) | 2 (15) |
| Grade 3/4 | 4 (2) | 4 (10) | 2 (9) | 0 (0) | 0 (0) | 1 (8) |
| Serious | 4 (2) | 4 (10) | 2 (9) | 0 (0) | 0 (0) | 1 (8) |
| Any death, n (%) | 129 (56)^†^ | 28 (68) | 14 (64) | 89 (77)^‡^ | 14 (78) | 11 (85) |
| AE | 54 (23)^†^ | 9 (22) | 6 (27) | 22 (19)^‡^ | 4 (22) | 3 (23) |
| Disease progression | 62 (27)^†^ | 15 (37) | 6 (27) | 53 (46)^‡^ | 9 (50) | 6 (46) |
| Other | 8 (3)^†^ | 2 (5) | 1 (5) | 6 (5)^‡^ | 0 (0) | 1 (8) |
| Unknown | 5 (2)^†^ | 2 (5) | 1 (5) | 8 (7)^‡^ | 1 (6) | 1 (8) |

*Some patients received both moderate and strong CYP3Ai. ^†^N=230. ^‡^N=115.
CYP3Ai, cytochrome P450 3A inhibitors.
